# Crafting work-nonwork balance involving life domain boundaries: Development and validation of a novel scale across five countries

**DOI:** 10.3389/fpsyg.2022.892120

**Published:** 2022-09-16

**Authors:** Philipp Kerksieck, Rebecca Brauchli, Jessica de Bloom, Akihito Shimazu, Miika Kujanpää, Madeleine Lanz, Georg F. Bauer

**Affiliations:** ^1^Public and Organizational Health, Center of Salutogenesis, Epidemiology, Biostatistics and Prevention Institute, University of Zurich, Zurich, Switzerland; ^2^Faculty of Social Sciences (Psychology), Tampere University, Tampere, Finland; ^3^Faculty of Economics and Business, University of Groningen, Groningen, Netherlands; ^4^Department of Policy Management, Keio University, Tokyo, Japan; ^5^School of Business, University of South-Eastern Norway, Hønefoss, Viken, Norway; ^6^Consumer Behavior Group, Institute for Environmental Decisions, ETH Zurich, Zurich, Switzerland

**Keywords:** life crafting, scale validation, work-nonwork balance, work-life balance, life domain interference, cross-cultural study

## Abstract

Ongoing developments, such as digitalization, increased the interference of the work and nonwork life domains, urging many to continuously manage engagement in respective domains. The COVID-19 pandemic and subsequent home-office regulations further boosted the need for employees to find a good work-nonwork balance, thereby optimizing their health and well-being. Consequently, proactive individual-level crafting strategies for balancing work with other relevant life domains were becoming increasingly important. However, these strategies received insufficient attention in previous research despite their potential relevance for satisfying psychological needs, such as psychological detachment. We addressed this research gap by introducing a new scale measuring crafting for a work-nonwork balance and examining its relevance in job-and life satisfaction, work engagement, subjective vitality, family role and job performance, boundary management and self-rated work-nonwork balance. The Work-Nonwork Balance Crafting Scale was validated in five countries (Austria, Finland, Germany, Japan, and Switzerland), encompassing data from a heterogeneous sample of more than 4,200 employees. In study 1, exploratory factor analysis revealed a two-factorial scale structure. Confirmatory factor analysis, test for measurement invariance, and convergent validity were provided in study 2. Replication of confirmatory factor analysis, incremental and criterion validity of the Work-Nonwork Balance Crafting Scale for job and life satisfaction were assessed in study 3. Study 4 displayed criterion validity, test–retest reliability, testing measurement invariance, and applicability of the scale across work cultures. Finally, study 5 delivered evidence for the Work-Nonwork Balance Crafting Scale in predicting work-nonwork balance. The novel Work-Nonwork Balance Crafting Scale captured crafting for the challenging balance between work and nonwork and performed well across several different working cultures in increasingly digitalized societies. Both researchers and practitioners may use this tool to assess crafting efforts to balance both life domains and to study relationships with outcomes relevant to employee health and well-being.

## Introduction

### Background and aim

Digitalization has led to the development of the world of work described as the “fourth industrial revolution” (Neufeind et al., 2018; Ropponen et al., 2020). An important aspect of this development is the increasing degree of freedom in individual work design and beyond. This freedom can be used by employees to shape their work individually and proactively through crafting (De Bloom et al., 2020; Tims et al., 2022). At the same time, more flexible work has lead to collapsing work-to-nonwork interfaces (Vaziri et al., 2020) and a vulnerable work-nonwork balance^1^ (WNB). Proactively crafting these interfaces and one’s work-nonwork balance constitute the core of this research.

The need for crafting WNB has mainly been fuelled by the trend to integrate work into other life domains due to: (a) the extended use of information and communication technologies (Piszczek, 2017), resulting in blurred boundaries between work and nonwork life domains (Ollier-Malaterre et al., 2019); (b) the demand for highly flexible work arrangements in a 24/7 economy (Bauer and Brauchli, 2017); and (c) organizational practices that encourage employees to expand work into nonwork life domains (Dumas and Sanchez-Burks, 2015). This trend is accompanied by increasing work density, which is the ratio of one’s workload over the resources available to perform that work (Derks and Bakker, 2014) and few opportunities for necessary recovery from work stress during and after work (De Bloom et al., 2015). In addition to the densification of work, demands in nonwork life domains are remaining or also increase (Rofcanin and Anand, 2020), leading to precarious situations for family life (Beckman and Mazmanian, 2020). Finally, the COVID-19 crisis, with mandatory home office regulations and lockdowns, has intensified this development (Cho, 2020; Vaziri et al., 2020; Rudolph et al., 2021). Proactive work designs including crafting are suggested as helpful strategies during this pandemic (Wang et al., 2020; Brauchli et al., 2022; Pijpker et al., 2022). Empirical evidence also highlights the importance of WNB for the well-being and health of employees (for reviews see Casper et al., 2018; Sirgy and Lee, 2018).

In summary, proactively balancing work and nonwork *via* crafting may comprise a beneficial behavioral strategy to improve WNB. To enable research and, later on, the dissemination of such proactive strategies, the present paper aimed to develop and validate the Work-Nonwork Balance Crafting (WNBC) scale.

### Defining the balance of work and nonwork

The first critical step in developing a new scale is defining the guiding concepts. This is demanding for WNB because currently, there is no consensus regarding guiding theoretical models and conceptual definitions available (Shockley et al., 2017; for an overview and review, see Casper et al., 2018; Wayne et al., 2021). Thus, we relied on Casper et al.’s (2018) thorough definition of WNB derived from a deductive, comprehensive literature review and dictionary classifications, as well as an inductive, qualitative analysis of employees’ definitions of such a balance:

“Employees’ evaluation of the favorability of their combination of work and nonwork roles, arising from the degree to which their affective experiences and their perceived involvement and effectiveness in work and nonwork roles are commensurate with the value they attach to these roles” (p. 197).

This definition incorporates the fit perspective to be satisfied with valued roles in their respective life domains (Greenhaus and Allen, 2011; Wayne et al., 2021). In addition, Casper et al. (2018) considered a current development in the WNB literature by exceeding the perspective of WNB as a balance between the domains of work and family only (Haar et al., 2019). Ideal balance is defined as a good commensuration between affective experiences and involvement and effectiveness in work and nonwork roles with value attached to these roles. To attain this fit, we proposed that individuals can engage in needs-oriented proactivity, the so-called crafting (De Bloom et al., 2020). The given WNB situation must not be passively accepted to satisfy these needs. Instead, we assumed that it can be beneficial if employees proactively adjust the WNB situation according to their own standards and respective role expectations [see Casper et al.’s (2018) WNB definition above]. For example, a father may adapt and therefore craft the balance of work and family life and, accordingly, his ideal WNB. Specifically, he might proactively craft his communication behavior at work by telling colleagues when he is unable to communicate with them during leisure time. Consequently, the development of our scale links the above WNB definition to the crafting concept considering an individual to be the effective agent of their WNB.

### The crafting approach as a point of origin for work-nonwork balance crafting: A brief review

Thus far, we described the relevance and development of WNB conceptually and outlined its relevance in the current world of work. In this section, we linked the origin of crafting with WNB to devise a new crafting construct beyond job crafting. Before we apply the crafting concept to the new domain of WNB crafting, we first review the well-established job crafting concept. Job crafting has been referred to as the self-initiated behaviors that employees take to shape, mold, and change their jobs (Wrzesniewski and Dutton, 2001; Tims and Bakker, 2010; Tims et al., 2012; Zhang and Parker, 2019). Crafting can help satisfying psychological needs and exhibits favorable outcomes, such as employee performance and well-being (for a review see Rudolph et al., 2017; Zhang and Parker, 2019; Mäkikangas and Schaufeli, 2021). Wrzesniewski and Dutton (2001) initially described this concept as a social constructivist approach that refers to “the physical and cognitive changes individuals make in the task or relational boundaries of their work” (p. 179). Wrzesniewski and Dutton distinguished three types of job crafting: (1) the changes employees make to adjust their work tasks (task crafting); (2) the quality and frequency of the relationships they have at work (relational crafting); and (3) the subjective meaning they assign to their work (cognitive crafting). These changes may be intentional and affect “the meaning of the work and one’s work identity” (Wrzesniewski and Dutton, 2001). This perspective on job crafting has inspired a large field of research because it helps, at least partially, overcome formal job constraints and invites new opportunities for individual work redesign.

Subsequently, Tims et al. (2012) integrated the crafting concept into the job demands resource (JD-R) model (Demerouti et al., 2001; Bakker and Demerouti, 2017) as a way to balance demands and resources and establish a person-job fit (Zhang and Parker, 2019). The JD–R model is a person-centred theoretical framework (Fan et al., 2019) and characterises crafting as a “self-initiated” and “self-targeted” individual-level strategy to increase person-job fit (Tims et al., 2012). Job demands (e.g., challenging and hindering demands) refer to aspects of a job that require sustained physical, emotional, or mental effort, whereas job resources (e.g., structural and social resources) refer to job aspects that stimulate personal growth and development while being functional in achieving work goals and simultaneously reducing job demands (Demerouti et al., 2001; Bakker and Demerouti, 2007; Lesener et al., 2019). Crafting, then, is defined as “the changes that employees may make to balance their job demands and job resources with their personal abilities and needs” (Tims et al., 2012). Extending the perspective on crafting in the light of resources and demands, Costantini et al. (2021) showed that employees can also restore the fit between their demands and preferences by optimising their demands instead of only decreasing them, see also Demerouti and Peeters (2018). This insight offers a new perspective to crafting research. Importantly, such demands optimizing crafting expands the work characteristics and tailors the work process to be more efficient by eliminating obstacles and simplifying procedures (Costantini et al., 2021). In summary, crafting includes the perspective that individuals can adapt their job to improve its fit to their abilities, needs, and preferences (Wrzesniewski and Dutton, 2001; Lichtenthaler and Fischbach, 2019).

The field has recently proceeded to transfer the concept of job crafting to life domains other than work, such as home crafting (Demerouti et al., 2019), off-job crafting (De Bloom et al., 2020), life crafting (Schippers and Ziegler, 2019), and leisure crafting (Berg et al., 2010; Petrou and Bakker, 2016). Crafting may also help achieve the requirements of modern work to nonwork arrangements. Accordingly, we assumed that crafting allows an individual to create an idiosyncratic balance of work and nonwork.

### Work-nonwork balance crafting concept underlying our scale

Our scale builds on a pioneering qualitative study by Sturges (2012) who defined crafting for WNB “as the unofficial techniques and activities that individuals use to shape their own work-life balance” (p. 1540). There, such crafting is characterized as self-initiated (Kossek and Ozeki, 1999), and goal-oriented behavior (Parker et al., 2010) proactively taking control over one’s WNB (Clark, 2000). Overall, it is driven by preferred role configurations in the respective life domains.

Sturges (2012) identified the three following crafting strategies for WNB, on which the development of our scale is established:

1. *Physical crafting* includes behaviors, such as time management, selection, and alternation of work location (e.g., leaving work early to do some necessary personal chores). The qualitative interviews indicated two subcategories: (a) *Temporal crafting* is about managing the time spent at work to achieve a WNB. An example here is finishing work on time, that is, adhering to contracted working hours. This factor might not entirely be the employee’s decision and necessarily be proactively negotiated with a supervisor, e.g., to avoid conflicts and synchronise work schedules. Therefore, temporal crafting may also involve relational crafting, which is outlined below. However, temporal crafting also refers to after-work time, that is, committing time to an event in the evening, such as sports or theatre. (b) *Locational crafting* is reported as occasionally choosing to work from home instead or in addition to working at the office. Thereby, locational crafting can help in accomplishing family chores or in reducing the strains of commuting. Note that these categories did not include *choosing a job* and *reducing travel time* by moving to live near work, since these factors are substantial life changes that go beyond typical crafting strategies that can vary day by day.

2. *Relational crafting* involves managing and using relationships at work and at home to secure and reinforce the kind of WNB that an individual wants to achieve (Sturges, 2012). Furthermore, relational crafting is structured in two sub-types: (a) *Managing out-of-work relationships* refers to socializing with people working on the same working times, which helps maintain their belief that one’s concept of WNB is typical. (b) *Managing work relationships* occurs by reducing unnecessary interactions at work and workloads; for example, when work is extended because the individual wants to reach a goal in the work domain, the quality of relations can be ensured by communicating this proactively to relevant persons in the work context, also management.

Finally, (3) *cognitive/emotional crafting* involves defining and framing the perceptions of what WNB means and entails (Sturges, 2012): *Conceptualization and the definition* of an idiosyncratic orientation and balance toward work and nonwork (e.g., meeting social engagements during the week despite regular long working hours), *prioritizing* work instead of the nonwork life domain (e.g., prioritizing work and highlighting the relevance of work-related achievements), and finally, *compromising* the ideal WNB to reach long-and short-term goals as a compromise in balancing both life domains, (e.g., investing long working hours for a sprint to reach a work-related achievement). We renamed Sturges’ (2012) dimension of cognitive crafting into cognitive/emotional crafting to integrate affective aspects as these aspects are particularly relevant for work to nonwork conflicts or enrichment (French et al., 2018; Wayne et al., 2022). Moreover, this component seems important as (role) balance has cognitive *and* affective elements (Casper et al., 2018). Besides the prioritization of work as orienting principle of one’s WNB we consequently add prioritizing non-work aspects for balancing both life domains. Further below, we outlined how the items of the WNBC scale align with these crafting techniques, see also Table 1.

**Table 1:** EFA factor structure.

Item		Factor loadings		
		WNBC-work	WNBC-nonwork	Crafting dimension
1	If I must get personal chores done during working time, I make sure that my work will not be negatively affected.	**0.46**	0.05	Cogn./Emot.
2	When I must get some work chores done, I come home later or go to work earlier, if necessary.	**0.79**	−0.22	Phys.
3	In some situations, I temporarily emphasize my work (e.g., work more before vacations to get things done).	**0.90**	−0.41	Cogn./Emot.
4	In certain phases of my life, I temporarily prioritize my work life to achieve a work goal.	**0.98**	−0.51	Cogn./Emot.
5	I try hard to meet my professional obligations, even if I’m demanded strongly by my private life.	**0.72**	−0.11	Cogn./Emot.
6	When I’m in a bad mood because of personal matters, I try not to let this affect my work environment.	**0.39**	0.14	Rela.
7	I make sure that I can enjoy the pleasant aspects of my work, even though I’m strongly demanded by my private life.	**0.54**	0.10	Rela./Cogn./Emot.
8	I tell people of my private environment when I’m unable to communicate with them during working time or to take care of private matters.	**0.48**	0.03	Rela.
9	If I must get work chores done during leisure time, I make sure that my personal life will not be negatively affected.	−0.20	**0.68**	Cogn./Emot.
10	When I must get some personal chores done, I come to work later or go home earlier, if necessary.	0.08	**0.41**	Phys.
11	In some situations, I temporarily emphasize my private life (e.g., when a friend needs my support).	−0.27	**0.75**	Cogn./Emot.
12	In certain phases of my life, I temporarily prioritize my private life to achieve a nonwork goal.	−0.38	**0.87**	Cogn./Emot.
13	I try hard to meet my private obligations, even if I’m demanded strongly by my work.	−0.06	**0.67**	Cogn./Emot.
14	When I’m in a bad mood because of work matters, I try not to let this affect my personal environment.	0.03	**0.45**	Rela.
15	I make sure that I can enjoy the time with my partner, my family or my friends even though I’m strongly demanded by my work.	−0.13	**0.79**	Rela./Cogn./Emot.
16	I tell people of my professional environment when I’m unable to communicate with them during leisure time or to take care of professional matters.	0.17	**0.34**	Rela.

Austrian, German, and Swiss sample in study 1, *N* = 320, factor loadings > 0.32 appear in bold. Cogn., cognitive; Emot., emotional; Rela., relational; Phys., physical.

### Adding crafting of the life domain boundary to work-nonwork balance crafting

Considering the eroding work-nonwork boundary (Allen et al., 2020; Vaziri et al., 2020), the balance, as well as the boundary, between the work-nonwork domains must be crafted.

The concept of boundary management (Sturges, 2008; Kossek et al., 2012) refers to the active shaping of boundaries. It is defined as a “construct that reflects our mental models about the permeability of the relationship between multiple life roles, our preferences about how to manage those relationships, and our choices and constraints regarding how we enact those preferences” (Rothbard and Ollier-Malaterre, 2015).

Boundary management practices are related to both interference and enhancement processes across life domains (Bulger et al., 2007). These boundary management practices are particularly linked to the successful integration of multiple important life roles (Rothbard et al., 2005; Kossek et al., 2021). Such life roles are crucial in the WNB definition used in our study (Casper et al., 2018; Vaziri et al., 2020) and are consequently important for WNBC. Research referring to the qualities of the boundaries between life domains typically defines core characteristics of the boundaries between work and other spheres of life that are relevant for WNBC. As such, *permeability* refers to the extent to which psychological and behavioral aspects can diffuse through the boundaries one has set (Ashforth et al., 2000; Clark, 2000). Second, *flexibility* means the contraction or expansion of a domain regarding its temporal and spatial constraints and is oriented toward requirements in either life domain (Hall and Richter, 1988). For example, if family chores are plenty, this allows extending the time spent within this life domain (e.g., leaving work early or reducing daily working time for care duties [flexibility] or answering calls from family members during working time [permeability]).

Moreover, the active configuration of work-nonwork boundaries is conceptualized on a continuum from segmentation to integration (Ashforth et al., 2000; Kossek and Lautsch, 2012; Wepfer et al., 2018). *Segmentation* refers to strict boundary-setting and inflexible and impermeable role boundaries. *Integration* is characterized by flexible and permeable role boundaries. Therefore, segmentation/integration characterizes the extent to which work and nonwork roles are separated.

Specifically, the nomological net for the item development of our WNBC is built on the work-home boundary theory (Ashforth et al., 2000). Ashforth et al. (2000) refer to roles that hold expectations, rules, and norms in respective life domains and converge with the conceptualizing of roles in the WNB definition by Casper et al. (2018), which underlies our WNBC scale. These roles and role transitions between life domains are characterized as psychological, physical, and temporal constructs. Thus, this theory aligns well with the physical and cognitive-emotional dimensions of Sturges’ (2012) WNBC techniques.

In the item development of the WNBC scale, we involved the proactive boundary management behaviors outlined in detail in the item description further below.

But here, we can summarize our conceptual basis of our scale development by providing the following complete definition underlying our scale: WNBC entails the unofficial techniques and activities individuals use to shape their own work nonwork balance under consideration of their boundary preferences and their favored combination of work and nonwork roles.

### Advantages over an earlier approach to measuring crafting a work-nonwork balance

Recently, Gravador and Teng-Calleja (2018) developed a work-life balance crafting behaviors survey. Like our scale, this instrument refers to Sturges (2012), but invokes a different theoretical framework. This instrument measures behaviors revolving around taking time off from work, fostering relationships with family and others, and working efficiently. The 25 items of the instrument are cumulated in eight clusters of proactive work-life balance crafting behavior themes (Gravador and Teng-Calleja, 2018).

Several shortcomings of this former approach are: First, Gravador and Teng-Calleja’s (2018) instrument contains the physical and relational crafting dimensions from Sturges’ (2012) concept, whereas the essential cognitive dimension is omitted. Cognitive crafting refers to framing and redefining WNB and shapes how employees view their WNB without engaging in specific behaviors. Therefore, we added this dimension to our scale. Second, Gravador and Teng-Calleja (2018) reported that only one of the eight scale dimensions, that is “working efficiently,” is associated with subjective well-being (satisfaction with life scale; Diener et al., 1985). In a second model, only two of the eight scale dimensions turned out to be related to work-life balance (work-life balance scale; Brough et al., 2014): “working effectively” and “saving private time.” These findings provide insights into the relative importance of crafting efforts but also shows that only few crafting dimensions of an extensive crafting scale matter. Accordingly, measuring crafting with fewer dimensions and items seems a more efficient way when examining links to well-being and WNB. Third, the instrument presented by Gravador and Teng-Calleja (2018) contains 25 items and eight clusters, and we determined the need for parsimonious instruments for, e.g., measurements in digital applications and online surveys. Besides the number of items, the complex structure and the high number of factors call for new scale development, building on the valuable results reported by Gravador and Teng-Calleja (2018). To the best of our knowledge, a WNBC scale that covers all three sub-dimensions proposed by Sturges (2012), that is, physical, relational, and cognitive/emotional crafting, has never been established. We aim to address this by developing the proposed new WNBC scale.

Moreover, three additional reasons are in favor of developing a new scale. First, the WNBC scale can potentially produce new opportunities for research by integrating two very productive and timely research streams, namely, WNB (Greenhaus and Callalan, 2020) and crafting (Hu et al., 2020). Second, our new scale can inform occupational health interventions, for which corresponding research and the development of an intervention are in progress. Third, we proposed a parsimonious two-factor structure of this scale, covering crafting in work and the nonwork life domains. Based on the presented considerations, our approach has advantages compared to previous approaches.

## Overview of the five studies for the development and validation of the work-nonwork balance crafting scale

To develop and validate the WNBC scale, we conducted a series of five studies. Studies 1, 2, 3, and 5 were conducted in German-speaking European countries, and study 4 relied on data from Finland and Japan. Study 1 involved generating and adapting items using expert reviews and exploratory factor analysis of the scale. Study 2 encompassed confirmatory factor analysis and assessed convergent validity, and measurement invariance across samples of study 1 and 2. In study 3, the incremental validity of the WNBC scale was studied and compared to the work-life indicator—a measure capturing work-nonwork function and the interplay of both life domains which is outlined further in detail below. The predictivity of the WNBC scale for work and life satisfaction was also assessed. Study 4 tested the scale’s criterion validity and test–retest reliability and provided initial evidence for the intercultural applicability of the WNBC scale. This study assessed the associations of the WNBC scale with work engagement, job performance, subjective vitality, and family role performance. Finally, study 5 involved measuring the relevance of WNBC for a global factor of WNB and the affective, effectiveness, and involvement dimensions of WNB.

In the following sections, we first presented the methods and results of each study separately. We then outlined and discussed the findings of these studies. Finally, the limitations and practical implications are presented.

## Study 1: Item development and factor identification

The development of an instrument that measures WLBC behaviors followed a stepwise approach. In the first step, we conducted comprehensive research of the relevant literature on crafting and another on the work-nonwork interface/balance. In particular, six instruments guided us in the development of new items: the *boundary enactment scale* (Wepfer et al., 2018), the *work-life crafting scale* (Peeters and Demerouti, 2014), the *job crafting scale* (Tims et al., 2012), the *work-life indicator* (Kossek and Lautsch, 2012), the *work-nonwork boundary strength scale* (Hecht and Allen, 2009), and the *SWING scale* (Geurts et al., 2005).

The existing scales inspired us regarding the proactive, self-initiated, and goal-oriented wording of the items. Based on this feature, we formulated new items in the second step. This procedure resulted in 37 items, which we grouped along with the theoretically assumed and from Sturges (2012) derived dimensions of “physical,” “cognitive/emotional,” and “relational” WNBC enacted both in the work and nonwork life domains. In the third step, we sent these items to seven experts in occupational health psychology and requested their comments.^2^ In addition, we asked laypersons to assess the comprehensibility and simplicity of the items. Given the feedback, the items were reworded and removed, and we confirmed that matching pairs of items for each life domain were constructed. In detail, one item captured crafting behaviors in the “physical,” “cognitive/emotional,” and “relational” dimensions in the work, and another matching item captured these respective WNBC in the nonwork life domain (see item outline below). Afterwards, we propose a parsimonious two-factor structure for this scale, representing the three WNBC efforts in the two life domains to be balanced. Setting up the scale with two factors representing each life domain will help in studying such crafting efforts with domain-specific antecedents and outcomes (Haar et al., 2019). Moreover, this scale structure will allow for measuring spillover effects across life domains (Wayne et al., 2022). For example, crafting for WNBC-nonwork may enhance processes that allow for and sustain recovery from work and, in turn, result in a better resource situation (e.g., better job performance, less work-related strain).

This procedure led to a preliminary pool of 32 items using a five-point Likert-type scale (1 = strongly agree, 5 = strongly disagree). Selecting a neutral scale mid-point is helpful because this answering format can be rescaled (Dawes, 2008), and it offers comparability with other scales in crafting research also used in this format (e.g., Tims et al., 2012).

Scree plots and other EFA procedures yielded no initial factor structure. We then applied the theory-driven model selection approach to the exploratory factor analysis (Preacher et al., 2013; Goretzko et al., 2019) for selecting the number of factors (m) that are maintained: “The role of theory in this process should be to determine, *a priori*, a set of plausible candidate models (i.e., values of m) that will be compared using observed data.” (Preacher et al., 2013). Based on our theoretical assumptions concerning the structure of this new scale, the following competitive factor solutions were tested: (a) a one-factor solution testing for a general WNBC factor; (b) a two-factor solution representing WNBC as a two-dimensional construct of work and nonwork; and (c) a three-factor solution representing the physical, cognitive/emotional, and relational WNBC as distinct factors. We tested these concurrent factor solutions since single-and three-factor solutions are prominent in relating crafting concepts beyond job crafting in the literature (see Demerouti et al., 2019 for a three-factor solution or Petrou and Bakker, 2016 for a single-factor solution).

*Research Question 1: Does the Work-Nonwork Balance Crafting Scale have (a) a one-factor structure representing physical, relational, and cognitive/emotional crafting in one general factor, (b) a two-factor structure representing crafting in the life domains of work and nonwork, or (c) a three-factor structure representing physical, relational, and cognitive/emotional crafting as distinct factors?*

*Hypothesis 1*: The Work-Nonwork Balance Crafting Scale displays satisfactory reliability in the derived factors.

### Methods

### Procedure and participants

The participants were recruited through an online panel data service in Austria, Germany, and Switzerland. All items were presented in German. Participation was voluntary and anonymous, and the confidentiality of their data was guaranteed. Persons who declared that they were under 18 years of age, unemployed, self-employed, or worked less than 9 h a week were not included. We excluded self-employed individuals because they represented a small and divergent group and because the social context at work is a relevant factor for crafting, even if crafting is a bottom-up strategy (Kerksieck et al., 2019; Tims and Parker, 2019). A total of 330 participants completed the questionnaire in April/May 2018. We used a *post hoc* multivariate outlier statistic to assess data quality controlled for Mahalanobis (1936) distance, which is also recommended for online studies (Niessen et al., 2016). The participants who answered the questionnaire in less than 5 min were classified as speeders (*N* = 7) and were excluded with multivariate outliers (*N* = 3), resulting in a sample size of 320 participants. The data were analyzed with SPSS 28.

Exactly half of the resulting sample was female. The average age of the participants was 43.96 years (*SD* = 12.11). Participants from Germany (63.3%), Austria (19.1%), and Switzerland (17.6%). A percentage of 46.3 worked 40–44 h per week. The average organizational tenure was 12.08 years (*SD* = 10.66). Most participants had completed vocational education (40.1%) or had a university degree (20.1%). The largest groups were employed in healthcare/social services (15%), public administration (12.2%), and commerce (10.3%).

### Results

#### Preparatory analysis

To assess potential common method bias (Podsakoff et al., 2012) in our self-reported data, we conducted a *post hoc* Harman single-factor test. An unrotated factor analysis revealed that the obtained factor accounted for 22.6% of the variance, suggesting that common method bias showed no pervasive effect on our data.

#### Exploratory factor analysis

To apply the model selection approach to exploratory factor analysis (Preacher et al., 2013; Goretzko et al., 2019), we used a criterion value of 0.32 to retain items (Costello and Osborne, 2005; Tabachnick and Fidell, 2014). Using oblimin rotation and Kaiser normalization (KMO = 0.774, *χ*^2^ = 1207.680, *df* = 120, Bartlett-test *p* < 0.001), we obtained a significant solution with 16 items in total and eight items in each of the two factors work and nonwork, explaining 37.63% of the variance. The alternative solution involving one factor provided lower amounts of explained variance (22.55%), whereas the three-factor solution (47.42%) did not yield a meaningful distribution of items aligning with these factors. Consequently, we derived a two-factorial structure of the WNBC scale, solving Research Question 1. The two factors were labelled “WNBC-work” and “WNBC-nonwork.” WNBC-work (-nonwork) refers to crafting one’s WNB, orienting efforts towards the life domain work (nonwork) according to one’s consideration of boundary preferences and favored combination of work and nonwork roles in a respective life domain.

Each factor contained eight items: one item covering physical crafting, four items covering cognitive/emotional crafting, and three items covering relational crafting, following the logic of Sturges’ (2012) qualitative analysis (Table 1).

The two dimensions demonstrated reliability above the recommended 0.70 level (Nunnally and Bernstein, 1994) with McDonald’s *ω* for WNBC-work = 0.75 and WNBC-nonwork = 0.71, confirming hypothesis 1.

#### Representation of crafting techniques in the WNBC scale

After determining 16 items to retain, we offer an item-by-item breakdown of how these items of the WNBC scale represent the crafting techniques identified in the qualitative study by Sturges (2012). We empirically derived two dimensions (work/nonwork). Both dimensions contain the crafting techniques referring to (a) *cognitive/emotional*, (b) *physical*, and (c) *relational crafting*. These three crafting techniques are equally represented in both scale dimensions, referring to work or nonwork. The following outline extends the item overview presented in Table 1, referring to crafting techniques and scale factors. Items 1 + 9 involve *cognitive/emotional crafting*, particularly the techniques of *prioritizing* one life domain “If I must get personal chores done during working time, I make sure that my work will not be negatively affected.” Items 3 + 11 likewise involve *cognitive/emotional crafting*; in detail, the technique of *prioritizing*: “In some situations, I temporarily emphasize my work (e.g., work more before vacations to get things done).” This *cognitive/emotional* crafting technique is also included in the following items that refer to the definition of an idiosyncratic WNB and *comprising* an ideal WNB. This may help for reaching goals in one of these life domains: Items 4 + 12 state “In certain phases of my life, I prioritize my work life in the meantime to achieve a work goal” and in items 5 + 13 read “I try hard to meet my professional obligations, even if I’m demanded strongly by my private life.” Focusing on emotional aspects of *cognitive/emotional crafting* and also involving *relational crafting* is reflected by items 7 + 15 “I make sure that I can enjoy the time with my partner, my family, or my friends even though I’m strongly demanded by my work.”

Items 2 + 10 relate to Sturges’ (2012) *physical crafting*, integrating both aspects of this dimension which are *temporal* and *locational crafting*: “When I must get some personal chores done, I come to work later or go home earlier, if necessary.”

In contrast, items 6 + 14 and items 8 + 16 refer to *relational crafting* aspects in the terminology of Sturges’ crafting techniques, since affect control helps to sustain positive relationships: “When I’m in a bad mood because of personal matters, I try not to let this affect my work environment” or “I tell people of my professional environment when I’m unable to communicate with them during leisure time or to take care of professional matters.”

We aimed to cover crafting efforts oriented toward the work-nonwork boundary in the following items. The contraction or expansion of a life domains (boundary flexibility) for tailoring boundaries toward requirements in either life domain is included in Items 2 + 10 “When I must get some work chores done, I come home later or go to work earlier, if necessary.” Items 6 + 14 involve proactively managing the boundary to prevent negative emotional life-domain spillovers: “When I’m in a bad mood because of work matters, I try not to let this affect my personal environment.” Items 8 + 16 state, “I tell people of my private environment when I’m unable to communicate with them during working time or to take care of private matters.” Here, the proactive boundary management strategy of segmentation and the prevention of permeability is applied to cover life domains from intruding and potentially disturbing communication across life domains.

## Study 2: Confirming factorial structure, measurement invariance, and convergent validity of the work-nonwork balance crafting scale

We investigated whether the factorial structure proposed in study 1 can be confirmed in this second study. We selected a single-and a three-factor solution as a concurrent factorial structure because these solutions can be derived from conceptual reasoning as outlined above. We used confirmatory factor analysis to test the following hypothesis:

*Hypothesis 2*: The two-factor solution of the Work-Nonwork Balance Crafting scale fits the data better than the alternative one-or three-factor solutions.

Moreover, invariance tests were performed for the psychometric properties of the assessed scale factors and their independence across samples 1 and 2.

*Hypothesis 3*: The Work-Nonwork Balance Crafting scale is invariant across the distinct samples in studies 1 and 2.

WNBC is defined as a proactive, self-initiated and goal-oriented individual-level, bottom-up approach. Consequently, WNBC is rooted in (a) personal initiative, which means that individuals take an active, self-starting approach to work and go beyond formal job requirements (Frese et al., 1997), and in (b) proactive personality, which is the relatively stable tendency to affect environmental change and is relatively unconstrained by situational factors (Bateman and Crant, 1993). Moreover, a proactive personality means taking initiative and action until a substantial change occurs (Raemdonck et al., 2017). Such traits are considered the underlying traits of job crafting (e.g., Bakker et al., 2012) and are assumed as such for WNBC. Therefore, proactive personality and personal initiative may drive the stability of WNBC over time and indicate convergent validity.

*Hypothesis 4a*: Both Work-Nonwork Balance Crafting scale dimensions correlate positively with personal initiative.

*Hypothesis 4b*: Both Work-Nonwork Balance Crafting scale dimensions correlate positively with proactive personality.

### Methods

#### Procedure and participants

Data were collected in April/May 2018 using the same procedure and inclusion criteria as that in study 1. Study 2 involved 324 new participants from Austria, Germany, and Switzerland. As previously mentioned, those who answered the questionnaire in less than 5 min (*N* = 8) and multivariate outliers were not included (*N* = 5), resulting in a sample size of 311 participants.

Sample 2 consisted of 57.6% male participants. The participants’ average age was 41.42 years (*SD* = 10.92). They lived in Germany (69.5%), Austria (18%), and Switzerland (12.5%). Half of the participants (47.9%) worked 40–44 h per week. On average, they had worked for 9.94 years (*SD* = 9.3) for their current employers. Most participants had completed vocational education (42.1%) or had a university degree (24.4%). The largest employment groups were employed in public administration (13.2%), commerce (11.9%), and the production of goods (8.4%).

#### Measures

WNBC was measured according to its subscales [see Table 1, scale parameters, including McDonald’s *ω* (Hayes and Coutts, 2020), are reported in Table 3], with items 1–8 representing WNBC-work and items 9–16 representing WNBC-nonwork. The participants rated the items using a five-point Likert-type scale with response options from 1 (totally disagree) to 5 (totally agree).

Personal initiative refers to active and self-induced behaviour beyond formal obligations in the workplace (Frese et al., 1997). Personal initiative was measured with a seven-item scale, with five-point Likert-type response options ranging from 1 (totally disagree) to 5 (totally agree). A sample item from the scale is “I use opportunities quickly to attain my goals.”

Proactive personality was measured with the six-item German translation of the proactive personality scale (Bateman and Crant, 1993). A sample item is “If I believe in an idea, no obstacle will prevent me from making it happen,” and it was answered on a five-point Likert-type scale with response options from 1 (totally disagree) to 5 (totally agree).

The items of the scales were translated into German and controlled by back-translation into the original English language.

### Results

#### Preparatory analysis

A Harman single factor test was computed to detect common method bias, and the results disclosed that the obtained single factor accounted for 21.1% of the variance, suggesting that common method bias was not present.

#### Confirmatory factor analysis

To conduct the confirmatory factor analysis, we tested three different factor models in unison with hypotheses 1a–1c in the EFA section. Confirmatory factor analysis was performed using SPSS AMOS 28. Table 2 shows the following indices for model fit assessment: comparative fit index (*CFI*), incremental fit index (*IFI*), root mean square error of approximation (*RMSEA*), and standardized root mean squared residual (*SRMR*). *CFI* and *IFI* must reach the cut-off value of 0.90 (Byrne, 2001), RMSEA < 0.06, SRMR < 0.08 (Hu and Bentler, 1999), and *χ*^2^/*df* ratio < 2 (Tabachnick and Fidell, 2014). Measurement residuals were correlated within and across latent constructs in an iterative process when significantly indicated and conceptually reasoned (e.g., for items 4 and 5, see Table 1).

**Table 2:** Fit statistics for confirmatory factor analyses and invariance tests.

Model	*χ* ^2^	*df*	CFI	IFI	SRMR	RMSEA
*CFA*						
Two-factor model	162.248	85	0.922	0.924	0.068	0.054
Three-factor model	266.194	85	0.816	0.822	0.083	0.083
One-factor model	267.883	86	0.815	0.822	0.084	0.083
*Invariance test*						
Model 1 (default model)	365.314	171	0.907	0.910	0.068	0.043
Model 2 (factor loadings constrained)	376.668	185	0.909	0.911	0.068	0.041
Model 3 (factor loadings and factor variances constrained)	380.905	187	0.908	0.910	0.070	0.041
Model 4 (factor loadings, factor variances, and covariances constrained)	402.800	205	0.906	0.907	0.071	0.039

CFI, comparative fit index; IFI, incremental fit index; SRMR, standardized root mean squared residual; RMSEA, root mean square error of approximation.

The one-factor model did not display a good fit (*χ*^2^ = 267.883, *df* = 86, *χ*^2^/*df* = 3.12, CFI = 0.815, IFI = 0.822, RMSEA = 0.083, SRMR = 0.084). The three-factor model did display an equally poor fit (*χ*^2^ = 266.194, *df* = 85, *χ*^2^/*df* = 3.13, CFI = 0.816, IFI = 0.822, RMSEA = 0.083, SRMR = 0.083). The goodness-of-fit indices of the two-factor model were good and superior (*χ*^2^ = 162.248, *df* = 85, *χ*^2^/*df* = 1.91, CFI = 0.922, IFI = 0.924, RMSEA = 0.054, SRMR = 0.068). In the two-factor model, all items loaded significantly on the matching latent variables (*p* < 0.01). In addition, the difference in the *RMSEA* approach with a 0.015 cut-off value as a method for determining the number of factors to retain was applied (Finch, 2020). Compared with the two-factor solution, both the one-factor and the three-factor models (∆RMSEA = 0.029), exceeded the recommended threshold. As the two-factor model best represented the data and outperforms the other models, hypothesis 2 was supported.

#### Invariance test

To test for the invariance of the WNBC scale, we performed a stepwise multigroup analysis (Byrne, 2004; Brown, 2015) with samples 1 and 2. These samples provide two distinct groups of participants. To some extent, this type of invariance testing resembles longitudinal invariance testing within the same sample, where time is the only distinct parameter (e.g., Spurk et al., 2011). So, we tested whether the scale functioned similarly in two different samples. We followed Cheung and Rensvold’s (2002) recommendation using the modelling approach and fit indices to verify the measurement invariance models. *CFI* differences (ΔCFI) lower than.01 were employed as the cut-off criteria. We further add fit indices to this test. In the first step, we tested the baseline model (model 1 in Table 2), in which all parameters were unconstrained. In the next step, model 2 with fixed factor loadings was compared and was invariant (∆CFI = 0.002, ∆IFI = 0.001, ∆SRMR = 0.000, ∆RMSEA = 0.002). Model 3, with additional constrained factor variances did not differ from the baseline model (∆CFI = 0.001, ∆IFI = 0.000, ∆SRMR = 0.002 ∆RMSEA = 0.002). In model 4, factor covariances were constrained additionally. The model was not different from the baseline model (∆CFI = 0.001, ∆IFI = 0.003, ∆SRMR = 0.003, ∆RMSEA = 0.004). The multigroup tests supported hypothesis 3, indicating measurement invariance across samples for the WNBC scale.

#### Convergent validity

To assess the convergent validity of the WNBC scale, we conducted analyses with personal initiative and proactive personality in samples 1 and 2. Table 3 shows partial correlations controlling for age, gender, education, and vocational position.

**Table 3:** Partial correlations and McDonald’s *ω* (between brackets on the diagonal) among the WNBC dimensions and personal initiative and proactive personality (controlled for gender, age, education level, and vocational position).

	*M*	*SD*	1	2	3	
*1. WNBC-work*						
Study 1	3.75	0.62	(0.75)			
Study 2	3.74	0.60	(0.72)			
*2. WNBC-nonwork*						
Study 1	3.48	0.61	0.25***	(0.71)		
Study 2	3.66	0.55	0.25***	(0.64)		
*3. Personal initiative*						
Study 1	3.80	0.60	0.49***	0.29***	(0.85)	
Study 2	3.80	0.64	0.46***	0.29***	(0.87)	
*4. Proactive personality*						
Study 1	3.65	0.61	0.43***	0.29***	0.75***	(0.84)
Study 2	3.65	0.62	0.43***	0.27***	0.80***	(0.83)

Austrian, German, and Swiss samples. Study 1: *N* = 320; study 2: *N* = 311.

M, mean; SD, standard deviation.

*p < 0.05; **p < 0.01; ***p < 0.001.

As hypothesized, positive correlations for personal initiative were found in sample 1 for WNBC-work (*r* = 0.49, *p* < 0.001) and WNBC-nonwork (*r* = 0.29, *p* < 0.001) as well as for sample 2 for WNBC-work (*r* = 0.46, *p* < 0.001) and WNBC-nonwork (*r* = 0.29, *p* < 0.001). Proactive personality correlated in sample 1 with WNBC-work (*r* = 0.43, *p* < 0.001) and WNBC-nonwork (*r* = 0.29, *p* < 0.001) as well as in sample 2 for WNBC-work (*r* = 0.43, *p* < 0.001) and WNBC-nonwork (*r* = 0.27, *p* < 0.001). Thus, hypotheses 4a and 4b were confirmed in both samples. These results indicate the convergent validity of the WNBC scale with constructs that are fundamentally associated with crafting.

## Study 3: Incremental validity of the work-nonwork balance crafting scale

We assessed the WNBC scale’s incremental validity compared to the work-life indicator (Kossek et al., 2012). The work-life indicator measures: (a) if work interrupts nonwork; (b) if nonwork interrupts work; (c) the perceived psychological control regarding the work-nonwork boundary and the degree of identification with, (d) a family role or (e) a work role. The WNBC scale and the work-life indicator share some similarities because both refer to work-nonwork function, and both scales focus on the interplay of both life domains. However, the work-life indicator determines *if* “(1) cross-role interruption behaviors (work into nonwork and nonwork into work); (2) identity centrality of work and family roles; and (3) perceived control of boundaries” (Kossek et al., 2012) are at stake. WNBC refers to *how* these interruptions are proactively arranged and integrated, considering the assumption of eroding work/nonwork boundaries that pressure WNB. Both concepts and scales are suitable for incremental validity testing by contrasting them on a WNB-related outcome, the satisfaction with job and life (Broeck et al., 2010; Haar et al., 2014). Therefore, we hypothesized:

*Hypothesis 5*: Work-Nonwork Balance Crafting at T1 predicts (a) job satisfaction and (b) life satisfaction at T2 above the work-life indicator.

### Methods

#### Procedure and participants

The procedure was the same as in studies 1 and 2. Participants were invited *via* an online panel data service. The sample consisted of employees from Austria, Germany and Switzerland, and the survey was conducted longitudinally with two measurement points at three-month intervals. Short time intervals were suggested by Dormann and Griffin (2015) for pilot panel studies. Gainfully employed individuals working more than 20 h per week and aged 18–65 years were included in the sample. Wave 1 represented 2,104 individuals; in wave 2, 1,502 (71.39%) individuals took part 3 months later. Data collections took place in December 2018 and March 2019. The surveys were conducted in the German language, and each scale was administered at each survey wave. Participants had a mean age of 43.68 years (*SD* = 11.13), and 50% were male. A percentage of 46 of the respondents reported working hours of 40–49 h per week. The sample represented a broad range of economic sectors and occupations, including health care and social work (13.5%), public administration (10.6%), and education (6.3%), and offered generalizability of the results.

### Measures

The WNBC scale was assessed with 16 items as developed in study 1 (Table 1). The reliability was McDonald’s *ω* = 0.67 for the WNBC-nonwork dimension and *ω* = 0.70 for the WNBC-work dimension. The WNBC scale was available in German, and other measures were translated from the published version in English to German and back-translated for accuracy.

The work-life indicator (Kossek et al., 2012) was assessed. Sample items read for the work interrupting nonwork subscale “I work during my vacations“, and for the nonwork interrupting work subscale “I do not think about my family, friends, or personal interests while working so I can focus“. Items were answered on a five-point scale ranging from *strongly disagree* to *strongly agree*. In our study, the subdimension’s reliability was *ω* = 0.80 for the work interrupting nonwork subscale and *ω* = 0.78 for the nonwork interrupting work subscale.

Life satisfaction and job satisfaction were measured using single-item measures adapted from Broeck et al. (2010): “How satisfied are you when you look at your private life as a whole?” and “How satisfied are you when you look at your professional life as a whole?” Both items were answered on a scale ranging from 1 (*extremely dissatisfied*) to 7 (*extremely satisfied*). Single-item measures are frequently used to assess general satisfaction (Lucas and Donnellan, 2012).

### Results

For the test of non-random sampling, we used multiple logistic regression (Goodman and Blum, 1996) while the dependent variable was coded dichotomously containing participants that either dropped out or participated in both study waves. The models included variables presented in the hypotheses (job/life satisfaction, work-life indicator). Nagelkerke (NK) *R*^2^ indicated that the explained variance in all estimated models was not substantial; therefore, none of the assessed variables indicated presence of non-random sampling: job satisfaction T1 (*B* = 0.04; *SE* = 0.03; *p* = 0.21; NK *R*^2^ = 0.00), life satisfaction T1 (*B* = 0.03; *SE* = 0.03; *p* = 0.43; NK *R*^2^ = 0.000), and work-life indicator T1 (*B* = −0.25; *SE* = 0.08; *p* < 0.001; NK *R*^2^ = 0.007).

**Figure 1:**
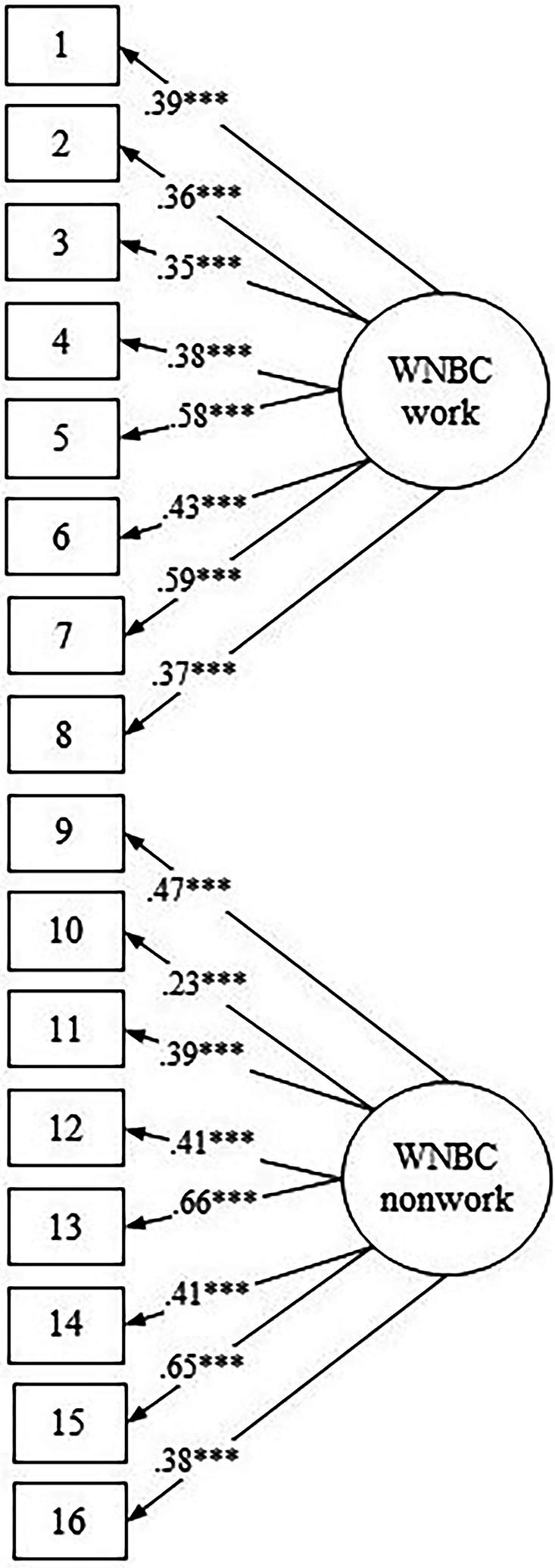
Configuration of WNBC scale with two-factorial CFA solution as presented in study 3. Latent constructs are shown in ellipses, and observed variables are shown in rectangles. Numbers in rectangles refer to WNBC scale item numbers, as presented in Table 1. ***p < 0.001.

Before hypothesis testing, we replicated the CFA of the two-factorial structure of the WNBC scale with the largest sample in this longitudinal study at wave 1 (*N* = 2,014): *χ*^2^ = 674.996, *df* = 85, CFI = 0.908, IFI = 0.909, RMSEA = 0.057, and SRMR = 0.062, see Figure 1.

When testing for incremental validity, the outcomes of life/job satisfaction at T2 were simultaneously regressed on the WNBC scale, the work-life indicator, and the respective outcomes (life/job satisfaction) at baseline T1. The results yielded by hierarchical linear regression analysis are shown in Table 4.

**Table 4:** Hierarchical regression analyses with predictors of job/life satisfaction.

	Life satisfaction T2
	*B*	SE *B*	*β*	*p*	Adj. *R*^2^	*ΔF*
*Step 1*						
Life satisfaction T1	0.512	0.021	0.532	< 0.001	0.283	
*Step 2*						
Life satisfaction T1	0.512	0.021	0.531	< 0.001	0.282	0.846 ns
Work-life indicator *Nwiw* T1	0.017	0.037	0.010	0.643		
Work-life indicator *Winw* T1	−0.048	0.038	−0.028	0.201		
*Step 3*						
Life satisfaction T1	0.499	0.021	0.517	< 0.001	0.296	15.973***
Work-life indicator *Nwiw* T1	−0.048	0.040	−0.029	0.227		
Work-life indicator *Winw* T1	−0.003	0.042	−0.002	0.949		
WNBC-nonwork T1	0.298	0.060	0.125	< 0.001		
WNBC-work T1	0.046	0.055	0.020	0.405		
	Job satisfaction T2					
*Step 1*						
Job satisfaction T1	0.513	0.021	0.525	< 0.001	0.275	
*Step 2*						
Job satisfaction T1	0.512	0.021	0.524	< 0.001	0.274	0.290 ns
Work-life indicator *Nwiw* T1	−0.015	0.038	−0.009	0.693		
Work-life indicator *Winw* T1	0.027	0.038	0.016	0.476		
*Step 3*						
Job satisfaction T1	0.504	0.022	0.515	< 0.001	0.280	6.657**
Work-life indicator *Nwiw* T1	−0.017	0.041	−0.010	0.680		
Work-life indicator *Winw* T1	0.001	0.042	0.001	0.981		
WNBC-nonwork T1	0.050	0.060	0.021	0.407		
WNBC-work T1	0.174	0.057	0.075	0.002		

Austrian, German, and Swiss samples in study 3 at T1: *N* = 2,104 and T2: *N* = 1,502.

Nwiw, nonwork interrupting work; winw, work interrupting nonwork.

*p < 0.05; **p < 0.01; ***p < 0.001.

Baseline-adjusted hierarchical linear regressions revealed that the WNBC scale explained the variance in two comprising and stable constructs above the work-life indicator (Table 4). In the first step, we included the respective variable at baseline (T1, accounting for a substantial amount of variance in the respective construct (job/life satisfaction) at T2). In the second step, the included work-life indicator did not add explained variance to the regression model. In step 3, the WNBC scale increased the amount of explained variance and improved the model significantly. In addition, such crafting showed a pattern of domain-specific predictors, confirming hypothesis 5.

## Study 4: Testing for criterion validity, measurement invariance and scale applicability across working cultures

In the fourth study, we investigated the criterion validity of the WNBC scale by the evaluating of associations with established constructs. At the same time, we tested the applicability of the WNBC scale in different countries and work cultures.

Leslie et al. (2019) indicated that variation in the relative importance of work and nonwork results from cultural values, such as in example, masculine societies, where individuals “live to work” (e.g., Japan) or” work to live” (e.g., Finland; Hofstede, 2011). Such cultural differences are relevant for WNB (Lewis and Beauregard, 2018). Given that WNBC is an individual-level proactive behavior, it may gain increased acceptance in individualistic cultures (Yang, 2005). In collectivistic cultures crafting for collective goods (e.g., family resources) can be accepted. By contrast, such individual strategies may be accepted if they are oriented toward positive outcomes for the community. Regarding work-related institutions and work-nonwork interference, Finland and Japan differ substantially in their cultural and institutional frameworks (Ollier-Malaterre et al., 2013) and regarding the resolution of work-to-nonwork conflicts, as shown by meta-analytical evidence where reports of work–family conflict were higher in collectivistic versus individualistic cultures (Allen et al., 2015). For an overview of the role of cultural values in crafting, see Kujanpää et al. (2021).

### Criterion validity and test–retest reliability

To test for criterion validity, we correlated the WNBC scale with external variables (Campbell, 1960) and used established constructs to test whether such crafting yields WNB-related outcomes. The validation criteria for the work dimension of the WNBC scale are work engagement and job performance. Subjective vitality and family role performance were evaluated as validation criteria for the nonwork dimension of the WNBC scale. We selected this set of variables to evaluate concepts related to crafting efforts for work-life balance that are directly linked to employee wellbeing and performance in both domains of life. Work engagement and job performance are frequently used to assess employee’s conditions in the work domain, also in combination with work-life balance (Johari et al., 2018; Wood et al., 2020). To mirror these concepts in the nonwork life domain, we assessed family role performance, since this concept relates well to both, performance in a typical nonwork domain is associated with WLB due to, for example caring duties but respective role expectations are also relevant in the WLB definition we are working with. Subjective vitality can be an important consequence of WLB promotion, such as sustainable careers (Kossek et al., 2014) and is transferred into both life domains as a relevant and dynamic reflection of well-being (Ryan and Frederick, 1997).

Moreover, we tested whether the WNBC scale is invariant and if these validation criteria hold across different countries and work cultures. Since the scale is developed first in German we use the largest German speaking sample as a reference. Finally, the test–retest reliability and the stability of associations with respective criteria over time were tested.

*Hypothesis 6*: The Work-Nonwork Balance Crafting Scale is invariant across samples from Austria, Germany, Switzerland, and Japan or Finland.

*Hypothesis 7a*: Work-nonwork Balance Crafting-work is positively associated with work engagement and job performance in Japan and Finland.

*Hypothesis 7b*: Work-nonwork Balance Crafting-nonwork is positively associated with family role performance and subjective vitality in Japan and Finland.

To test the assumption that WNBC has substantial and time-stable effects on respective outcomes, we hypothesized the following:

*Hypothesis 8*: The Work-Nonwork Balance Crafting Scale displays test–retest reliability after 3 months in Japan and Finland.

*Hypothesis 9a*: Work-Nonwork Balance Crafting-work at T1 is positively associated with work engagement and job performance at T1 and T2 in Japan and Finland.

*Hypothesis 9b*: Work-Nonwork Balance Crafting-nonwork at T1 is positively associated with family role performance and subjective vitality at T1 and T2 in Japan and Finland.

### Methods

#### Procedure and participants

Items in the WNBC scale were translated by professional translation agencies and back-translated by bilingual individuals of the research team for the Finnish and Japanese surveys. The back translations were then compared with the German source versions for consistency. All other scales were translated from the published English versions. We involved the largest German-speaking sample (as described in study 3) for invariance testing.

Data were collected in longitudinal studies in Finland (starting September 2018) and Japan (starting December 2018), each with two measurement waves (3 months apart). Again, each scale was administered at each survey wave. Participation was voluntary and anonymous, and the confidentiality of participants’ data was guaranteed. The participants were at least 18 years old and worked a minimum of 24 h per week. The Finnish sample included data from 357 individuals in the first wave and 221 individuals in the second wave after a three-month interval; most of whom were female (85.2%). Participants were workers mostly recruited through HR staff mainly from the public sectors. A total of 38 participants from an earlier study agreed to participate, and 70 were recruited through social media. The average age of the participants was 49.7 years (*SD* = 10.2). On average, they worked 38.9 (*SD* = 4.4) hours per week and had worked for 14.7 years (*SD* = 11.9) for their current employers. Furthermore, 26.9% had completed a bachelor’s degree, and 19.9% had a master’s degree. The largest employment groups worked in social and healthcare (37.1%) and public administration (19.6%).

The Japanese sample contained data from 204 individuals in the first wave and 128 individuals in the second wave, among which 63.2% were male. Participants were recruited through a consultancy agency working with various Japanese companies. The mean age was 31.9 years (*SD* = 6.4). The participants worked for 48.4 h (*SD* = 9.6) per week and had worked for 4.9 years (*SD* = 4.6) for their current employers. In addition, 78% held a bachelor’s degree, and 59.3% worked in the IT sector.

#### Measures

WNBC was measured according to its subscales, as described above. The scale parameters are reported in Table 6.

Job performance was measured with the following World Health Organization work performance questionnaire item (Kessler et al., 2003): “How would you rate your work performance within the past month on a scale from 1 to 10, where 1 is the worst job performance anyone could have at your job, and 10 is the performance of a top worker?” The time reference was adapted to 1 month.

Work engagement referring to a positive work-related state of fulfillment was measured with the nine-item version of the Utrecht work engagement scale (Schaufeli et al., 2006), containing the dimensions of vigor (e.g., “At my work, I feel bursting with energy”) and dedication (e.g., “I find the work that I do full of meaning and purpose”), adapted to a retrospection of 1 month. The response scale comprised 1 = never, 2 = once per month, 3 = a few times per month, 4 = once a week, 5 = a few times a week and 6 = daily.

Family role performance assesses the performance within the family domain and depended on the balance of work-nonwork roles; it was assessed by the family role performance scale (Chen et al., 2014). This scale consists of eight items referring to the fulfilment of several role expectations in family life. The Likert-type scale for this item ranged from 1 = did not fulfil expectations at all to 5 = fulfilled expectations completely. Furthermore, the time reference was changed to 1 month: “To what extent do you think you fulfilled what was expected of you in relation to the following aspects of your current family life over the past month?”

Subjective vitality is “the experience of having positive energy available to or within the regulatory control of one’s self” (Ryan and Frederick, 1997), in contrast to being driven or compelled. A four-item instrument measured this concept (Bostic et al., 2000), and a five-point Likert-type scale with response options ranging from 1 (not at all) to 5 (very true) was used. The retrospective reference frame was adapted to 1 month.

### Results

#### Preparatory analysis

The Harman single-factor test was executed, indicating that the attained factor in the Japanese sample accounted for 22.8% of the variance, and the single factor in the Finnish sample accounted for 25.8% of the variance. This result showed that no common method bias was present in our data. Data were analysed using SPSS 28 and SPSS AMOS 28.

#### Measurement invariance

For testing hypotheses 6, multigroup CFAs test four increasingly strict levels of invariance, as Vandenberg and Lance (2000) outlined. The first model is an unconstrained model (configural invariance). The second model tests the invariance of factor loadings (metric invariance), residuals (residual variance), and intercepts (scalar invariance). Results for both series of invariance testing between the largest sample from German-speaking countries and samples from Japan or Finland indicated acceptable to good fit indices (Table 5). We followed Cheung and Rensvold’s (2002) approach using fit indices to verify the measurement invariance and used a stepwise approach as suggested by Putnick and Bornstein (2016). Differences in fit indices lower than 0.01 were used as the cut-off criteria. First, invariance testing across the German-speaking and Japanese samples indicated configural, and metric invariance due to differences fit indices (*∆* < 0.01). Scalar invariance was not indicated, partially confirming hypothesis 6 across the German-speaking and Japanese samples. Second, invariance testing across the German-speaking sample and the sample from Finland showed likewise configural and metric invariance. Also, scalar invariance was not indicated here, partially confirming hypothesis 6 across the German-speaking sample and the sample from Finland. Summing up, we found results showing configural, metric measurement invariance but not scalar measurement invariance in both series of invariance testing for the WNBC scale across respective countries and work cultures.

**Table 5:** Fit statistics for invariance tests across countries.

	*χ* ^2^	*df*	*p*	CFI	IFI	RMSEA (90% CI)	SRMR	∆ CFI	∆ IFI	∆ RMSEA	∆ SRMR
*Austria, Germany, Switzerland, vs. Japan*											
Configural invariance	160.148	91	<0.001	0.990	0.991	0.018 [0.013; 0.023]	0.020	–	–	–	–
Metric invariance	193.676	105	<0.001	0.988	0.988	0.019 [0.015; 0.023]	0.021	0.002	0.003	0.001	0.001
Scalar invariance	634.795	121	<0.001	0.929	0.930	0.043 [0.040; 0.046]	0.020	0.059	0.058	0.024	0.001
*Austria, Germany, Switzerland, vs. Finland*											
Configural invariance	207.395	105	<0.001	0.986	0.987	0.020 [0.016; 0.024]	0.022	–	–	–	–
Metric invariance	264.343	119	<0.001	0.981	0.981	0.023 [0.019; 0.026]	0.023	0.005	0.006	0.003	0.001
Scalar invariance	722.400	135	<0.001	0.922	0.923	0.043 [0.040; 0.046]	0.024	0.059	0.058	0.20	0.001

#### Criterion validity

The results of the criterion validity test for the dimensions of the WNBC scale are reported in Table 6. The WNBC-*work* dimension was positively correlated with job performance in the Finnish sample (*r* = 0.24, *p* < 0.001) and the Japanese sample (*r* = 0.24, *p* < 0.01). They were also significantly correlated with work engagement in the Finnish sample (*r* = 0.35, *p* < 0.001) and the Japanese sample (*r* = 0.49, *p* < 0.001). These significant correlations of WNBC-*work* with job performance and work engagement supported hypothesis 7a.

**Table 6:** Partial correlations and McDonald’s *ω* between brackets on the diagonal (T1/T2) among the WNBC dimensions, job performance, work engagement, family performance and subjective vitality (controlled for gender, age, education level and vocational position) in the sample from Finland and Japan.

	*M* T1	*SD* T1	*M* T2	*SD* T2	1	2	3	4	5	6
1. WNBC-work/Finland	4.07	0.57	4.12	0.50	(0.59/0.58)					
WNBC-work/Japan	3.86	0.57	4.00	0.49	(0.67/0.67)					
2. WNBC-nonwork/Finland	3.77	0.65	3.77	0.65	0.40***	(0.69/0.65)				
WNBC-nonwork/Japan	3.76	0.57	3.77	0.57	0.32***	(0.68/0.75)				
3. Job performance/Finland	7.99	1.30	8.12	1.16	0.24***	0.24***	(single item)			
Job performance/Japan	5.89	2.06	6.02	2.16	0.24**	0.17*				
4. Work engagement/Finland	4.59	1.22	4.54	1.21	0.35***	0.25***	0.53***	(0.95/0.94)		
Work engagement/Japan	4.07	1.30	4.14	1.36	0.49***	0.03	0.29***	(0.95/0.95)		
5. Family role performance/Finland	3.80	0.75	3.84	0.69	0.12*	0.31***	0.31***	0.26***	(0.85/0.84)	
Family role performance/Japan	3.12	0.91	3.09	1.01	−0.02	0.28***	0.25**	0.06	(0.88/0.91)	
6. Subjective vitality/Finland	3.56	0.90	3.46	0.85	0.21***	0.32***	0.41***	0.06***	0.42***	(0.93/0.94)
Subjective vitality/Japan	3.33	1.07	3.43	1.10	0.23**	0.15*	0.36***	0.69***	0.14	(0.95/0.96)
	*Study variables at T2 Finland*									
7. WNBC-work T1/Finland							0.16**	0.25***	0.13*	0.23***
8. WNBC-nonwork T1/Finland							0.22***	0.21***	0.30***	0.28***
	*Study variables at T2 Japan*									
9. WNBC-work T1/Japan							0.12	0.38***	0.04	0.26**
10. WNBC-nonwork T1/Japan							0.25**	0.14	0.35***	0.28**

Finish sample at T1: *N* = 357 and T2: *N* = 221; Japanese sample at T1: *N* = 204 and T2: *N* = 128, as in study 4.

M, mean; SD, standard deviation

*p < 0.05; **p < 0.01; ***p < 0.001.

The WNBC-*nonwork* subscale was positively correlated with family role performance in the Finnish sample (*r* = 0.31, *p* < 0.001) and the Japanese sample (*r* = 0.28, *p* < 0.001). It was additionally positively correlated with subjective vitality in the Finnish sample (*r* = 0.32, *p* < 0.001) and the Japanese sample (*r* = 0.15, *p* < 0.05). These significant correlations of WNBC-*nonwork* with family role performance and subjective vitality confirmed hypothesis 7b.

#### Test–retest reliability

Hypothesis 8 was confirmed as the WNBC-*nonwork* dimension at T1 was positively correlated (*p* < 0.001) at T2 within the same sample (Japan, *r* = 0.59 or Finland, *r* = 0.67). This result was also consistent for the WNBC-*work* dimension, which was positively correlated (*p* < 0.001) from T1 to T2 within the Japanese sample (*r* = 0.63) and within the Finnish sample (*r* = 0.62). All partial correlations were controlled for gender, age, education level, and vocational position.

To test the stability and long-term effect of WNBC, we correlated both scale dimensions for work and nonwork with outcome variables with an interval of three-months. Partial correlations were controlled for gender, age, education level, and vocational position. In Finland, WNBC-*work* at T1 was significantly correlated with job performance at T2 (*r* = 0.16, *p* < 0.01) and work engagement at T2 (*r* = 0.25, *p* < 0.001), confirming hypothesis 9a. WNBC-*nonwork* at T1 was significantly correlated with family role performance at T2 (*r* = 0.30, *p* < 0.001) and subjective vitality at T2 (*r* = 0.28, *p* < 0.001), confirming hypothesis 9b.

In Japan, WNBC-*work* at T1 was not significantly correlated with job performance at T2 (*r* = 0.12, *ns*), but it was significantly correlated with work engagement at T2 (*r* = 0.38, *p* < 0.001), partially confirming hypothesis 9a. WNBC-*nonwork* at T1 was positively correlated with family role performance at T2 (*r* = 0.35, *p* < 0.001) and with subjective vitality at T2 (*r* = 0.28, *p* < 0.01), confirming hypothesis 9b.

## Study 5: Testing WNBC on an essential outcome

In the fifth study, we elaborated on WNB as an essential outcome of the WNBC scale. To measure WNB, we used the recently published WNB scale (Wayne et al., 2021).

Wayne et al. (2021) provided a four-dimensional scale involving a distinct (1) *global balance dimension* referring to “employees’ appraisals of how they combine work with nonwork roles,” where the attitude object is the “combination of work and nonwork roles.”

Further three dimensions are (2) *affective balance*, which is defined as “the perception that one experiences sufficiently pleasant emotions in work and nonwork roles commensurate with the value attached to those roles,” (3) *effectiveness balance*, which is “the perception that one’s effectiveness in work and nonwork roles is commensurate with the value attached to the roles;” (4) and *involvement balance* is “the perception that one’s involvement in work and nonwork roles is commensurate with the value attached to the roles.”

To assess the associations of the WNBC scale with the dimensions of the WNB scale, we formulated the following hypotheses:

*Hypothesis 10*: Work-nonwork Balance Crafting is positively associated with the global level of work-nonwork balance.

Specifically, work-nonwork balance crafting is positively associated with the (a) effectiveness balance, (b) affective balance, and (c) involvement balance dimensions of work-nonwork balance.

### Methods

#### Procedure and participants

The data were collected *via* an online panel data service in a cross-sectional study in Austria, Germany, and Switzerland in November 2021. Participation was voluntary and anonymous, and the confidentiality of participants’ data was guaranteed. The participants were at least 18 years old and worked a minimum of 20 h per week. A percentage of 46.1 reported working 46-49 h per week. The sample included *N* = 924 individuals; 43.9% were female and had an average age of 48.87 years (*SD* = 10.1). 34.9% had completed a university/applied university degree, and 43.9% had vocational education. Moreover, 4.3% completed primary education, and 16.9% completed high school as the highest educational degree. Data analysis was conducted using the lavaan package of R Project for Statistical Computing 09.02 build 382.

#### Measures

WNBC was measured in German according to its subscales, which are described above. The reliability was McDonald’s *ω* = 0.71 for the WNBC-nonwork dimension and *ω* = 0.74 for the WNBC-work dimension. Measurement residuals were correlated within and across latent constructs.

To measure WNB, we used Wayne et al.’s scale (2021). This scale was translated from English to German (the survey language) and was back-translated into English. This scale includes four subdimensions, as outlined and defined above. A sample item for *global balance* reads: “Overall, my work and nonwork roles fit together” (*ω* = 0.91). Further scale dimensions and sample items are for *involvement balance* “I am able to be adequately involved in the work and nonwork roles that matter most to me” (*ω* = 0.88); for the *effectiveness balance*: “I am able to effectively handle important work and nonwork responsibilities” (*ω* = 0.88), and for the *affective balance*: “I experience a lot of positive emotions in my most highly valued work and nonwork roles” (*ω* = 0.92). Items were rated on a five-point Likert scale 1 = *strongly disagree* to 5 = *strongly agree*.

### Results

Before testing the full structural equation model (SEM), we provide a CFA of the WNB scale (Wayne et al., 2021). To our knowledge, this study is the first to apply this WNB scale in the German language (*χ*^2^ = 557.360, *df* = 164, CFI = 0.972, TLI = 0.968, SRMR = 0.030, and RMSEA = 0.051). A SEM for the association of the new WNBC scale dimensions and a WNB applying the scale for measuring such balance by Wayne et al. (2021) indicated the relevance of crafting for WNB (Table 7). For each of the WNB subdimensions, the WNBC scale delivered significant results and accounted for substantial variance in the outcome: WNBC-work was positively associated with WNB global balance (*β* = 0.48, *p* < 0.001), WNB involvement (*β* = 0.37*, p* < 0.001), WNB effectiveness (*β* = 0.56*, p* < 0.001) and WNB affective (*β* = 0.38, *p* = 0.001). Accordingly, the dimension of WNBC-nonwork was positively associated with the WNB global balance (*β* = 0.31, *p* = 0.006), with WNB involvement (*β* = 0.43*, p* < 0.001), WNB effectiveness (*β* = 0.37*, p* < 0.001) and WNB affective (*β* = 0.38, *p* < 0.001). The parameters for the model fit of the SEM indicated good fit (*χ*^2^ = 1621.431, *df* = 566, CFI = 0.943, TLI = 0.937, RMSEA = 0.045, and SRMR = 0.050). Thus, hypotheses 10 and 10a–c were confirmed.

**Table 7:** Structural equation model assessing WNBC on work-nonwork balance (Wayne et al., 2021) dimensions.

	*β*	SE *B*	*p*	*R* ^2^
WNB *global balance*				
WNBC-work	0.48	0.14	<0.001	0.22
WNBC-nonwork	0.31	0.11	0.006	
WNB *involvement*				
WNBC-work	0.37	0.11	<0.001	0.35
WNBC-nonwork	0.43	0.10	<0.001	
WNB *effectiveness*				
WNBC-work	0.56	0.11	<0.001	0.41
WNBC-nonwork	0.37	0.10	<0.001	
WNB *affective*				
WNBC-work	0.38	0.11	0.001	0.27
WNBC-nonwork	0.38	0.10	<0.001	
*Model parameters*				
*χ*^2^ = 1621.431, *df* = 566, CFI = 0.943, TLI = 0.937, SRMR = 0.050, RMSEA = 0.045				

CFI, comparative fit index; TLI, Tucker Lewis index; SRMR, standardized root mean squared residual; RMSEA, root mean square error of approximation.

## Overall discussion of findings across studies

We conducted this series of studies to develop a new tool that measures crafting efforts employees exert to achieve a WNB that is in line with an individual’s needs and standards. The WNBC scale captures a new concept and a new, cross-cutting domain of crafting, expanding the fruitful research streams of job crafting as well as crafting in the nonwork life domain, such as home crafting (Demerouti et al., 2019), life crafting (Schippers and Ziegler, 2019), and off-job crafting (De Bloom et al., 2020). Conceptually, our scale development was established on the crafting behaviors identified in a pioneering qualitative study conducted by Sturges (2012). Instead of studying crafting in the work and nonwork domains separately, the WNBC scale aims to grasp how employees can craft an idiosyncratic *balance* of work and nonwork life under consideration of their favored boundaries and combination of their work and nonwork roles. We expect that such crafting supports attaining a WNB and related outcomes, including employee wellbeing.

### Development of items and implementation of subdimensions

In study 1, we developed the scale’s items by building on earlier conceptual development and expert feedback. The following exploratory factor analysis yielded a two-factorial structure with eight items in each scale dimension—one capturing crafting on aspects of the *work*-life domain, and the other on aspects of the *nonwork*-life domain. Both scale dimensions cover physical, relational, and cognitive/emotional WNBC. We consider this parsimonious two-factor structure of the WNBC scale to be an advantage compared with earlier approaches for measuring work-life balance crafting, containing eight clusters (Gravador and Teng-Calleja, 2018). In addition, our new scale contained the previously omitted cognitive dimension identified by Sturges’ (2012) study and extended this dimension, including important emotional aspects.

### Test of competing factorial solutions of the work-nonwork balance crafting scale

Furthermore, in study 2, the confirmatory factor analysis revealed that a two-factor model reached good fit indices, outperforming the one-, three-and six-factor models. Additionally, a test for measurement invariance between the samples of studies 1 and 2 indicated that the WNBC scale was robust across several measurement occasions. Further, the WNBC scale dimensions correlated positively with proactive personality and personal initiative, displaying convergent validity to proactivity—a core crafting element (Bakker et al., 2012).

Interestingly, WNBC-work showed a higher correlation with proactive personality and personal initiative than WNBC-nonwork. Seemingly, proactivity and initiative-taking on a trait level were expressed considerably concerning the life domain of work. This concept may be explained by the fact that WNBC at work occurs in a highly formalized and hierarchical context with the informal, proactive private environment, thus requiring more proactivity to overcome the formal constraints of crafting this domain. This finding offers a new avenue for future research to extend the existing knowledge of personality traits for WNB crafting. This call was additionally fuelled by recent research on personality traits and crafting (Oprea et al., 2019) and should involve research on gender roles that may manifest in WNB decisions (Adamson et al., 2022).

### Incremental validity evidence

The results of study 3 indicated the incremental validity of the WNBC. While WNBC was predicting job/life satisfaction above the work-life indicator, the latter was not significantly predicting these outcomes in any conducted analyses. Crafting for WNB was significantly associated with increased satisfaction in both life domains. This finding was compelling since many other contributing factors drive life/job satisfaction (Heller et al., 2004), explaining why added explained variance is not large *per se*. This is mainly due to the variety of stable factors (Ilies et al., 2019) relevant to life and job satisfaction.

We assumed that we could explain job and life satisfaction variance beyond the work-life indicator because we combined both crafting the WNB and boundaries in a meaningful way. This strategy was done because WNBC goes beyond merely allowing or preventing life domain transfers as suggested in the strategies of segmentation or integration (e.g., Bulger et al., 2007). The WNBC scale added crafting techniques for qualitatively shaping these transfers and respective role transitions. Because of increasingly blurred boundaries and working from home regulations before and during the COVID-19 pandemic, resulting in increased work-nonwork interference, it was important taking these boundaries more into account. Both developments resulted in less physical and time-bound boundaries (Allen et al., 2020; Vaziri et al., 2020), making WNBC an essential behavioral strategy.

### Applicability across several different working cultures

Study 4 involved data from German-speaking countries, Finland, and Japan, which tested for invariance, criterion validity, test–retest reliability, and intercultural applicability of the WNBC scale. Analyses for invariance across these countries indicated that the WNBC provides metric measurement invariance. The absence of scalar invariance can be subject to different contextual factors across cultures, specifically concerning invariance testing across Japan, Austria, Germany, and Switzerland. However, strict invariance is challenging to achieve in heterogeneous groups (Clench-Aas et al., 2011) and may therefore be difficult to reach in cross-cultural research. Such measurement variance may occur due to differences in (work) cultures (Zhou et al., 2019), as has also been shown in research on proactivity and WNB (Smale et al., 2019). For example, in comparing a Chinese sample with a British or Spanish sample, Nielsen et al. (2017) showed a lack of factor loading invariance in the job crafting questionnaire potentially caused by cultural differences. Nevertheless, crafting scales may provide meaningful results in various countries, but cultural comparisons should be conducted cautiously (Schachler et al., 2019). Cultural differences may also be related to work-life balance, as discussed below and may thus affect cross-cultural invariance testing.

Analysing data from the Japanese and Finish samples showed correlations with external criteria of job performance (Kessler et al., 2003), work engagement (Schaufeli et al., 2006), family role performance (Chen et al., 2014), and subjective vitality (Ryan and Frederick, 1997) were positive at the cross-sectional level. Moreover, positive associations between WNBC and these concepts can be found in longitudinal data from Finland and Japan, indicating the relevance and stability of WNBC outcomes. Beyond this generalizability of the scale to different cultural contexts, the WNBC scale can unravel differences related to work culture. The WNBC scale indicated measurement variance for scalar measurement invariance across work cultures, which can be interpreted in a way that this scale seems sensitive to cultural differences in WNBC. Thus, the WNBC scale offers a measure for capturing such differences, but for comparing such crafting across countries, respective cultural differences need to be considered (Zhou et al., 2019). This is indicated in systematic variation of findings across countries: Individuals exerting WNBC-nonwork reported increased family role performance and subjective vitality in Finland. A result supported by the theoretical underpinning of Leslie et al. (2019) and Hofstede (2011), referring to Finland as a highly feminist work culture. In Finland, cultural norms concerning work and nonwork roles may support crafting for a WNB focusing on the nonwork domain. This finding also supported the compensation hypothesis (Beigi et al., 2019), according to which undesired states in one domain were compensated for in another life domain, as shown, for example, for leisure crafting (Petrou and Bakker, 2016). Besides, WNBC-nonwork correlated with work engagement in the sample from Finland but not in Japan. WNBC in the nonwork life domain may restore resources in Finnish participants that they were able to transfer to engagement in the work domain. This concept is an effect that has been studied in the context of sustainable careers (Kelly et al., 2020) and to which the WNBC scale can add further knowledge in future research.

### Association with core outcome work-nonwork balance

Finally, in study 5, on another set of more than 900 employees, we inquired how WNBC efforts cross-sectionally contributed to the outcome of balancing work and nonwork. Both the work and nonwork dimensions of the WNBC scale were associated with the balance of both life domains on a global unidimensional and multidimensional formative construct (Wayne et al., 2021). Interestingly, employees’ appraisal of how well they combine work with nonwork roles on the global construct was positive in individuals who crafted their WNB regarding the work domain. The WNB of our European sample may be more affected by the work domain since they assign more relative importance to this life domain (see Leslie et al., 2019 for work priority beliefs). Therefore, (a) crafting work in comparison to nonwork may be more important to arrive at a positive evaluation of the “combination of work and nonwork roles” and (b) crafting the work domain can lead to satisfaction with individual standards that focus more on work-related achievements (Kelliher et al., 2019). However, crafting the nonwork life domain also substantially contributed to a positive global evaluation of WNB. In detail, crafting both life domains led to favorable judgments of items such as “Overall, my work and nonwork roles are integrated” and “My work and nonwork roles are combined in ways that are harmonious.”

This reasoning may also explain why WNBC-work contributed more to a positive evaluation of *effectiveness balance*. Crafting for WNB, focusing on work-related aspects, helped employees arrive at a highly proactive role balance. Performance and successfulness as key terms in these items may have spurred the link to crafting in the work domain because this domain is perceived as more performance-based (Kim, 2014). In addition, Wayne et al. (2021) suggested that work or family design factors (e.g., the significance of these life domains) can determine how performance in either life domain is judged. This factor may have contributed to the relative importance of work-related crafting aspects. However, crafting in both life domains was relevant for a positive appraisal of WNB effectiveness.

*Involvement balance* was strongly associated by WNBC-nonwork. Since salient work-related demands that call particularly for role involvement in the work domain for many employees may exist, WNBC-nonwork helped conclude a positive appraisal regarding the desired balance of role involvement in *both* life domains. This perspective on WNB would fit in with the “expandable-pie” perspective, stating that involvement in one role expands (i.e., enriches) resources for another role (Leslie et al., 2019; Rothbard et al., 2021). Further research is necessary to understand the role of WNBC in enrichments or even gain cycles, as has already been found for *job* crafting (Vogt et al., 2016). Nevertheless, crafting in both life domains was again relevant for balanced involvement in both life domains.

Our analysis indicated that crafting for WNB in both life domains was similarly associated with *affective balance*. Showing WNBC efforts led to a more positive affective balance and, therefore, more positive and fewer negative emotions in highly valued roles across life domains. This finding was important since Marks and MacDermid (1996) outlined that role balance involves affective and cognitive elements. The results indicated that the WNBC scale can capture proactive efforts that improve both the cognitive (involvement/effectiveness) and affective dimensions of balance.

The results of study 5 were in favor of the WNBC scale, as it captured crafting efforts for attaining a WNB. Here, we demonstrated that crafting for WNB, as measured with this new crafting scale, explains significant variance in employee WNB. A broad set of antecedents (e.g., work/family demands and resources) relevant for the combined study on crafting and WNB offers a variety of new research questions and outcomes (e.g., work-family interference) to be studied with the WNBC scale.

In summary, the validity and potential of the WNBC scale were displayed by extensive testing in several samples across different work cultures. Our scale is the first rigorously developed scale for measuring the construct of WNBC. As such, it has great potential to advance the scientific study of crafting the vital concept of WNB, which has elicited much attention and scholarship (Rothbard et al., 2021). The need for the study of WNBC is amplified by the increasing tendencies of blurred boundaries between life domains. This trend is exacerbated by COVID-19 measures and telework (Kniffin et al., 2020), making the balance of various life roles throughout the day an essential topic for many. The WNBC scale takes blurred boundaries into account while orienting crafting towards either life domain, as reflected in the two-factor structure of this measure. We hoped that WNBC could help employees establish a sustainable, resourceful WNB.

### Practical implications

Given the current economic developments and work regulations imposed during the COVID-19 pandemic, employees’ WNB is increasingly under pressure (Kniffin et al., 2020). Organizations can encourage WNBC to improve their employees’ quality of life in both life domains. Our results indicated that WNB crafting is relevant for the sake of employee vitality, family role/job performance, job/life satisfaction, work engagement and self-reported WNB. This feature is important because crafting as measured with the WNBC scale illustrates behaviors that may support employee health and well-being. Crafting allows employees to purposefully balance their resources and demands of work and nonwork by proactively balancing both life domains.

For implementation in organizations, the training and education of supervisors and employees involved these crafting behaviors are relevant for employee WNB. Especially with increasing job demands, a decrease in WNB has been observed, which has been counterbalanced by supervisor support and job autonomy (Haar et al., 2019), both of which are relevant for crafting. Since WNBC can be performed at the individual level, this approach is also available to individuals without organizational support. Nevertheless, given that crafting can be trained (Gordon et al., 2018), organizations must foster opportunities for WNBC and train employees to craft their WNB. A web-based intervention (application) is currently being developed for such training purposes. Results derived from studies involving the WNBC scale will inform this application.

### Limitations and future research

Besides the strength of this study, several important limitations must be acknowledged.

First, the internal consistency of WNBC subscales is relatively low in several of our validation studies. However, we cover a fairly broad spectrum of crafting efforts oriented towards WNB (physical, cognitive/emotional, relational crafting) in a compact scale with relatively few items. This implies that both sub-dimensions of the WNBC scale (work/nonwork) represent three crafting behaviours. Thus, the modest reliability coefficients seem to reflect that items were chosen to represent this conceptual breadth within the WNBC construct rather than to maximize internal consistency, likewise prominently implemented elsewhere (Ryff and Keyes, 1995). Therefore, internal consistency can be expected to be low. In fact, we would argue that internal consistency is a questionable criterion for scale quality. Adding highly similar items will lead to high internal consistency. But the additional items will add little information regarding the underlying construct (Boyle, 1991) while increasing participant burden. Our scale captures a broad spectrum of crafting efforts with a compact scale with relatively few items. To provide a more robust measure of internal consistency, we reported McDonalds *ω* (Zinbarg et al., 2005). Given the relatively low reliability and that items map different aspects of WNWB, future research may investigate whether items of the WNBC are formative or reflective of the construct (Coltman et al., 2008).

Second, the applied cut-off values for several CFAs model-fit assessment referring to WNBC are above standard cut-off values (Byrne, 2001) but below the.95-threshold. In this regard, we refer to an ongoing debate on the so-called “golden rules” for cut-off criteria (Niemand and Mai, 2018), while future research may apply more strict factor analytical criteria. Nevertheless, we would like to recommend our scale in the presented factor structure for use in research and practice. We base this recommendation on the convincing results of our studies, particularly study 5, which shows a substantial association between our new scale captured crafting efforts for WNB and the actual WNB measured (Wayne et al., 2021).

Third, the median age differed between the Finnish and Japanese samples, which could have biased our results. Persons identifying as female were overrepresented in the Finnish sample, and persons identifying as male were overrepresented in the Japanese sample. This imbalance in gender distribution may have aggravated the differences between countries since Finland is known to have a highly feminist work culture. Therefore, age and gender were added as control variables in the analyses. These sample characteristics may have been one reason for the lack of measurement invariance across countries for the WNBC scale. We took a first step in comparing the WNBC scale across cultures, providing evidence for metric invariance [for a comparable outline in job crafting research, see Nielsen et al. (2017)].

Fourth, we studied data from five countries with diverse cultural backgrounds that may have influenced the results (Ollier-Malaterre and Foucreault, 2017). At this point, the systematic variations and differences between the studied countries were not further analyzed. Examine the cultural variation in WNBC in further detail is beyond the scope of this validation paper. Future studies may include the interplay of cultural norms with WNBC.

In general, current perspectives on crafting need to be extended by broadening the research focus to areas of life beyond work, as done in this paper.

## Conclusion

Approaching a balance of work and nonwork according to individual needs and standards has gained relevance under increasingly demanding work-nonwork conditions (Greenhaus and Callalan, 2020). With the WNBC scale, we contributed a new and useful tool for crafting research. In doing so, we stimulate future research on two constructs that gained high practical and research interest: Work-nonwork balance and crafting.

Presented findings indicate that the WNBC scale is relevant for outcomes in both life domains, such as job-and life satisfaction, work engagement, subjective vitality, family role and job performance, work-life boundary management, and self-rated WNB. The applicability of this new scale and the importance of its findings in a variety of occupational settings and work cultures are displayed.

We outlined the many opportunities to link this scale with productive research streams such as research on personality, work culture, work/family interference, work-nonwork balance, and work arrangements due to COVID-19 regulations that call for new ways of balancing life domains. Thus, we hope this scale spawns new research and informs interventions for aiding individuals using this proactive crafting approach to establish their WNB.

## Data availability statement

The raw data supporting the conclusions of this article will be made available by the authors, without undue reservation.

## Ethics statement

Ethical review and approval was not required for the study on human participants in accordance with the local legislation and institutional requirements. The patients/participants provided their written informed consent to participate in this study.

## Author contributions

PK: conceptualization, methodology, formal analysis, investigation, data curation, and writing—original draft. RB: conceptualization, methodology, formal analysis, investigation, data curation, and writing—review and editing. JB: conceptualization, investigation, and writing—review and editing. AS, MK and ML: investigation and writing—review and editing. GB: conceptualization, methodology, investigation, writing—review and editing, and funding acquisition. All authors contributed to the article and approved the submitted version.

## Funding

The University of Zurich Foundation supported data collection in Germany, Austria, Switzerland and the contribution of PK, GB, and RB. The Swiss National Science Foundation supported further data collection and the contribution of PK and GB (grant number: SNF 100019M_201113). Part of the data collection and the work of JB were supported by the Academy of Finland (grant number: 308718).

## Acknowledgments

We thank Sylvia Broetje, Luisa Grimm, Michaela Knecht, Laurenz L. Meier, Wilmar Schaufeli, Hiroyuki Toyama, and Dana Unger for their expert feedback, suggestions, and support throughout this research project.

## Conflict of interest

The authors declare that the research was conducted in the absence of any commercial or financial relationships that could be construed as a potential conflict of interest.

## Publisher’s note

All claims expressed in this article are solely those of the authors and do not necessarily represent those of their affiliated organizations, or those of the publisher, the editors and the reviewers. Any product that may be evaluated in this article, or claim that may be made by its manufacturer, is not guaranteed or endorsed by the publisher.

## Footnotes

^1^In the following sections of this paper we use the term work-nonwork balance to indicate that work and life are not necessarily distinct entities but can be better structured in work and nonwork life domains [see also Wayne et al. (2021)].

^2^We would like to thank Sylvia Broetje, Luisa Grimm, Laurenz Meier, Dana Unger and Wilmar Schaufeli for their comments on earlier version of this scale.
